# Sialic acid utilization by *Cronobacter sakazakii*

**DOI:** 10.1186/2042-5783-3-3

**Published:** 2013-05-24

**Authors:** Susan Joseph, Sumyya Hariri, Naqash Masood, Stephen Forsythe

**Affiliations:** 1 Pathogen Research Centre, School of Science and Technology, Nottingham Trent University, Clifton Lane, Nottingham NG11 8NS, UK

**Keywords:** *Cronobacter sakazakii*, Sialic acid utilisation, Sialidase, Virulence factor

## Abstract

**Background:**

The *Cronobacter* genus is composed of seven species, and can cause infections in all age groups. Of particular concern is *C. sakazakii,* as this species is strongly associated with severe and often fatal cases of necrotizing enterocolitis and meningitis in neonates and infants. Whole genome sequencing has revealed that the *nanAKT* gene cluster required for the utilisation of exogenous sialic acid is unique to the *C. sakazakii* species (ESA_03609–13).

Sialic acid is found in breast milk, infant formula, intestinal mucin, and gangliosides in the brain, hence its metabolism by *C. sakazakii* is of particular interest. Therefore its metabolism could be an important virulence factor. To date, no laboratory studies demonstrating the growth of *C. sakazakii* on sialic acid have been published nor have there been reports of sialidase activity. The phylogenetic analysis of the *nan* genes is of interest to determine whether the genes have been acquired by horizontal gene transfer.

**Results:**

Phylogenetic analysis of 19 *Cronobacter* strains from 7 recognised species revealed the *nanAKTR* genes formed a unique cluster, separate from other *Enterobacteriaceae* such as *E. coli* K1 and *Citrobacter koseri*, which are also associated with neonatal meningitis. The gene organisation was similar to *Edwardsiella tarda* in that *nanE* gene (N-acetylmannosamine-6-phosphate-2epimerase) was not located within the *nanATK* cluster. Laboratory studies confirmed that only *C. sakazakii*, and not the other six *Cronobacter* species, was able to use sialic acid as a carbon source for growth. Although the ganglioside GM1 was also used as carbon source, no candidate sialidase genes were found in the genome, instead the substrate degradation is probably due to β–galactosidase activity.

**Conclusions:**

Given the relatively recent evolution of both *C. sakazakii* (15–23 million years ago) and sialic acid synthesis in vertebrates, sialic acid utilization may be an example of co-evolution by one species of the *Cronobacter* genus with the mammalian host. This has possibly resulted in additional virulence factors contributing to severe life-threatening infections in neonates due to the utilization of sialic acid from breast milk, infant formula, milk (oligosaccharides), mucins lining the intestinal wall, and even gangliosides in the brain after passing through the blood–brain barrier.

## Background

The *Cronobacter* genus is an emergent group of bacterial pathogens in the *Enterobacteriaceae* family. The majority of infections (bacteraemia, and urinary tract infections) are in the adult population, however the most publicized cases are severe, and frequently fatal infections in neonates and infants [1,2]. In these highly vulnerable populations, the organism is associated with necrotizing enterocolitis and a highly destructive form of meningitis in which the bacterium crosses the blood–brain barrier and causes abscess formation in the brain cavity [3,4]. The genus is composed of seven species, and multilocus sequence typing has been used to describe the diversity of the genus [5,6]. Evolutionary analysis suggests that the *C. sakazakii* species separated from the rest of the *Cronobacter* genus 15–23 million years ago (MYA) [6]. Recent whole genome studies have revealed that *C. sakazakii* is the only *Cronobacter* species that has the *nanAKT* gene cluster encoding for sialic acid utilization [7,8]. Since sialic acid is found in breast milk, infant formula, mucin lining the intestinal tract and gangliosides in the brain [9], it is plausible that this metabolism may account for the predominance of *C. sakazakii* in neonatal and infant infections. However no laboratory studies investigating this trait have been undertaken to date.

Sialic acid can exist in nearly 50 different forms with the most studied being 2-keto-3-deoxy-5-acetamido-D-glycero-D-galacto-nonulosonic acid, often abbreviated as Neu5Ac. This sialic acid is generally found bound to sugars to form polysaccharides, and also can be bound to lipids or proteins to form sialo-glycoconjugates. With few exceptions, sialic acid conjugates are notably absent from many eukaryotic lineages, including most protostomes, plants, fungi, and protists. It is postulated that sialic acid synthesis evolved in animals and later emerged in bacterial pathogens and commensals either by convergent evolution or horizontal gene transfer. A number of microbial strategies have evolved which target host sialic acids for adherence, mimicry, and degradation [10,11]. Some bacteria can produce sialidase (or neuraminidase), encoded by *nanH,* to cleave sialic acid from the glycoconjugate forms. This gene has low homology (<30%) across bacterial groups, and has not been described in many organisms [12,13]. Although neonatal meningitic *E. coli* K1 is able to grow on sialic acid, it lacks the enzyme sialidase. However it is possibly able to obtain sufficient sialic acid from the activities of other sialidase-producing bacteria in the environment or from the host cells expressing the enzyme in conditions of inflammation [10,13].

The uptake of sialic acid through the outer cell membrane of Gram negative bacteria is by an outer membrane porin, NanC. There are three different types of transporters for the inner membrane: NanT, a major facilitator superfamily (MFS) protein; TRAP, a tripartite ATP-independent periplasmic transport system; and an ATP-binding cassette (ABC) transporter. All members of the *Enterobacteriaceae* studied to date have shown the presence of the single-component NanT transport system [10,11,14]. Once transported into the cell, the Neu5Ac lyase (NanA) converts sialic acid (Neu5Ac) into N-acetylmannosamine (ManNAc) and phosphoenolpyruvate (PEP). NanK is an ATP-dependent kinase specific for ManNAc generating N-acetylmannosamine-6-phosphate (ManNAc-6-P). ManNAc-6-P epimerase (NanE) then converts the ManNAc-6-P into N-acetylglucosamine-6-phosphate (GlcNAc-6-P). GlcNAc-6-P deacetylase (NagA) and glucosamine-6-P deaminase (NagB) convert GlcNAc-6-P into fructose-6-phosphate, which is a substrate in the glycolytic pathway. NanR is the repressor that regulates the activity of these genes. The genes for the first three enzymes (*nanA, nanK* and *nanE*) are usually found clustered together forming the *nan* gene cluster [11]. However, there have been a few exceptions such as *Citrobacter freundii* and *Edwardsiella tarda* where the *nanE* gene is located in a separate region from the rest of the operon [14]. The genes encoding NagA and NagB are located adjacent to each other, but most often not necessarily in the vicinity of the *nan* gene cluster on the bacterial genomes [12-14]. The genes within the cluster show independent evolutionary histories. Several horizontal gene transfer events are noted in the phylogenetic trees for all three proteins. Most significantly, the NanA protein shows several possible horizontal gene transfer events between the Eukaryotes and Prokaryotes. Two examples are the clustering of *Trichomonas vaginalis* NanA protein sequences with *Pasteurellaceae*, and the *Bacteroides*, *Yersinia* and *Vibrio* branching closely with the Eukarya kingdom [11,14].

The uptake of sialic acid into bacterial cells has been associated with a number of virulence factors. The bacterial glycolipid capsule is an example of molecular mimicry of the host as it aids the organism to overcome the immune responses of the host. Neonatal meningitic *E. coli* K1 uses sialic acid to decorate the cell surface, and *Cronobacter* does produce capsular material, especially when grown on milk [15]. Sun *et al*. [16] reported the O-PS gene for *C. turicensis* G3882 included N-acetylneuraminic acid synthetase and CMP-N-acetylneuraminic acid synthetase. However the accurate identification of this strain is uncertain as the 16S rRNA gene sequence (Accession no. HQ880409) does not match other strains of *C. turicensis*.

As previously reported for the genome sequenced strain *C. sakazakii* BAA-894, the gene cluster encoding for a putative sugar isomerase (YhcH) and the *nanKTAR* genes (encoding N-acetylneuraminate and N-acetylmannosamine degradation) are located at ESA_03609-13 [7,8]. However, the remaining *nan* genes for sialic acid metabolism have not been described in detail, and these previous bioinformatic studies did not identify any candidate sialidase (NanH) genes [8].

*C. sakazakii* is associated with infections of low birth weight neonates, and this could be linked to a number of opportunities for the organism to utilize sialic acid for growth. Human milk contains sialic acid in the form of sialyloligosaccharides which are highest in colostrum and decreases by nearly 80% over the following 3 months after birth [9,17]. Human milk from mothers of preterm infants contains 13–23% more sialic acid than milk from mothers of full-term infants at 3 of the 4 lactation stages. Similarly infant formulas contain sialic acid which may be bound to glycoproteins. Although the nutritional significance of sialic acid is unknown, it is plausible that it contributes to sialic acid accumulation in the brain. Breast milk also contains a variety of sialoglycans; secretory IgA, lactoferrin, and oligosaccharides. Human milk oligosaccharides are poorly digested and are substrates for intestinal bacterial metabolism, promoting bacterial growth in the intestinal tract. As a site of bacterial attachment, the intestinal microvilli of neonates have increased sialic acid and N-acetylglucosamine residues whereas adults have increased mannose, glucose, and fucose residues [17,18]. Finally, the brain is the major site of sialic acid display in the form of gangliosides (sialylated glycolipids) and sialic acid may have a role in the structural and functional establishment of synaptic pathways [9]. It is possible that, like *Neisseria meningitidis*, *Streptococcus pneumoniae* and *Haemophilus influenzae* which cause bacterial meningitis in children <5 years, that *C. sakazakii* has a developmental dependence on access to the central nervous system.

Given the common clinical association of *C. sakazakii* infection of neonates with necrotising enterocolitis and brain damage, an improved understanding of sialic acid utilisation by the organism was warranted. This paper describes the plausible link between the recent evolution of sialic acid metabolism by *C. sakazakii* and its pathogenicity as well as re-investigates the possible presence of sialidase activity.

## Results

### Distribution of sialic acid utilization genes

The distribution of sialic acid metabolism genes in *Cronobacter* and other related *Enterobacteriaceae* is shown in Table 1. Essentially *nanAKRTC* and *yhcH* were only found in the genomes of *C. sakazakii* and none of the other *Cronobacter* species. These genes were also found in *E. coli* K1 strain CE10 and *Cit. koseri*, and were not in the related *Enterobacteriaceae* members *Pantoea* spp. or *E. cloacae*. *NeuC* encoding for UDP-N-acetylglucosamine 2-epimerase was found in all strains. The genes *neuA, neuB, neuD, neuO* and *neuS* were absent from all the *Cronobacter* spp. genomes. *NeuA* and *neuB* were only found in *E. coli* K1 strain CE10.

**Table 1:** **Distribution of the sialic acid utilisation and other related genes across the sequenced genomes of the *Cronobacter *genus**

**Gene**	**Loci**	**Function**	***C. sakazakii *BAA-894**	***C. sakazakii *680**	***C. sakazakii *701**	***C. sakazakii *E899**	***C. sakazakii *696**	***C. malonaticus *507**	***C. malonaticus *681**	***C. turicensis *564**	***C. turicensis z*3032**	***C. universalis *581**	***C. muytjensii *530**	***C. dublinensis *582**	***C. dublinensis *1210**	***C. condimenti *1330**
*yhcH*	ESA_03609	Putative sugar isomerase	**+**	**+**	**+**	**+**	**+**	**-**	**-**	**-**	**-**	**-**	**-**	**-**	**-**	**-**
*nanK*	ESA_03610	N-acetylmannosamine kinase	**+**	**+**	**+**	**+**	**+**	**-**	**-**	**-**	**-**	**-**	**-**	**-**	**-**	**-**
*nanT*	ESA_03611	Sialic acid transporter (permease)	**+**	**+**	**+**	**+**	**+**	**-**	**-**	**-**	**-**	**-**	**-**	**-**	**-**	**-**
*nanA*	ESA_03612	N-acetylneuraminate lyase	**+**	**+**	**+**	**+**	**+**	**-**	**-**	**-**	**-**	**-**	**-**	**-**	**-**	**-**
*nanR*	ESA_03613	Transcriptional regulator	**+**	**+**	**+**	**+**	**+**	**-**	**-**	**-**	**-**	**-**	**-**	**-**	**-**	**-**
*nanC*	ESA_03302	N-acetylneuraminic acid outer membrane channel protein	**+**	**+**	**+**	**+**	**+**	**-**	**-**	**-**	**-**	**-**	**-**	**-**	**-**	**-**
*nanE*	ESA_00529	N-acetylmannosamine-6-phosphate 2-epimerase	**+**	**+**	**+**	**+**	**+**	**+**	**+**	**+**	**+**	**+**	**+**	**+**	**+**	**+**
*nagA*	ESA_02662	N-acetylglucosamine-6-phosphate deacetylase	**+**	**+**	**+**	**+**	**+**	**+**	**+**	**+**	**+**	**+**	**+**	**+**	**+**	**+**
*nagB*	ESA_02661	Glucosamine-6-phosphate deaminase	**+**	**+**	**+**	**+**	**+**	**+**	**+**	**+**	**+**	**+**	**+**	**+**	**+**	**+**
*neuC*	ESA_03772	UDP-N-acetylglucosamine 2-epimerase	**+**	**+**	**+**	**+**	**+**	**+**	**+**	**+**	**+**	**+**	**+**	**+**	**+**	**+**
*siaPQM*	ESA_04264-4266	tripartite ATP-independent periplasmic (TRAP) transporter	**+**	**+**	**+**	**+**	**+**	**+**	**+**	**+**	**+**	**+**	**+**	**+**	**+**	**+**

### %GC content of nan genes

As a possible indicator of horizontal gene transfer events the %GC content of the sialic acid utilization genes was calculated and compared with the rest of the *Cronobacter* genome. The *nanA, nanR* and *nanT* genes of the five *C. sakazakii* genomes showed average GC% values of 57.2%, 56.32% and 57.14% respectively which is similar to the average 56% GC content of the *Cronobacter* genomes. In contrast, *nanE* and *nanK* showed slightly higher values of 63% and 62.2% respectively, whereas *nanC* had a much lower%GC content value of 47.4%. This was also found for the *nanC* gene in the closely related organisms *Cit. koseri* (48%), *E. cloacae* (51%), *Enterobacter* sp. (44.54%), and *E. coli* K1 (43%). The genomes of these organisms are between 55-58% GC. The *nagA* and *nagB* genes showed GC% values of 56.3% and 54% respectively.

### Phylogenetic analysis

**Figure 1:**
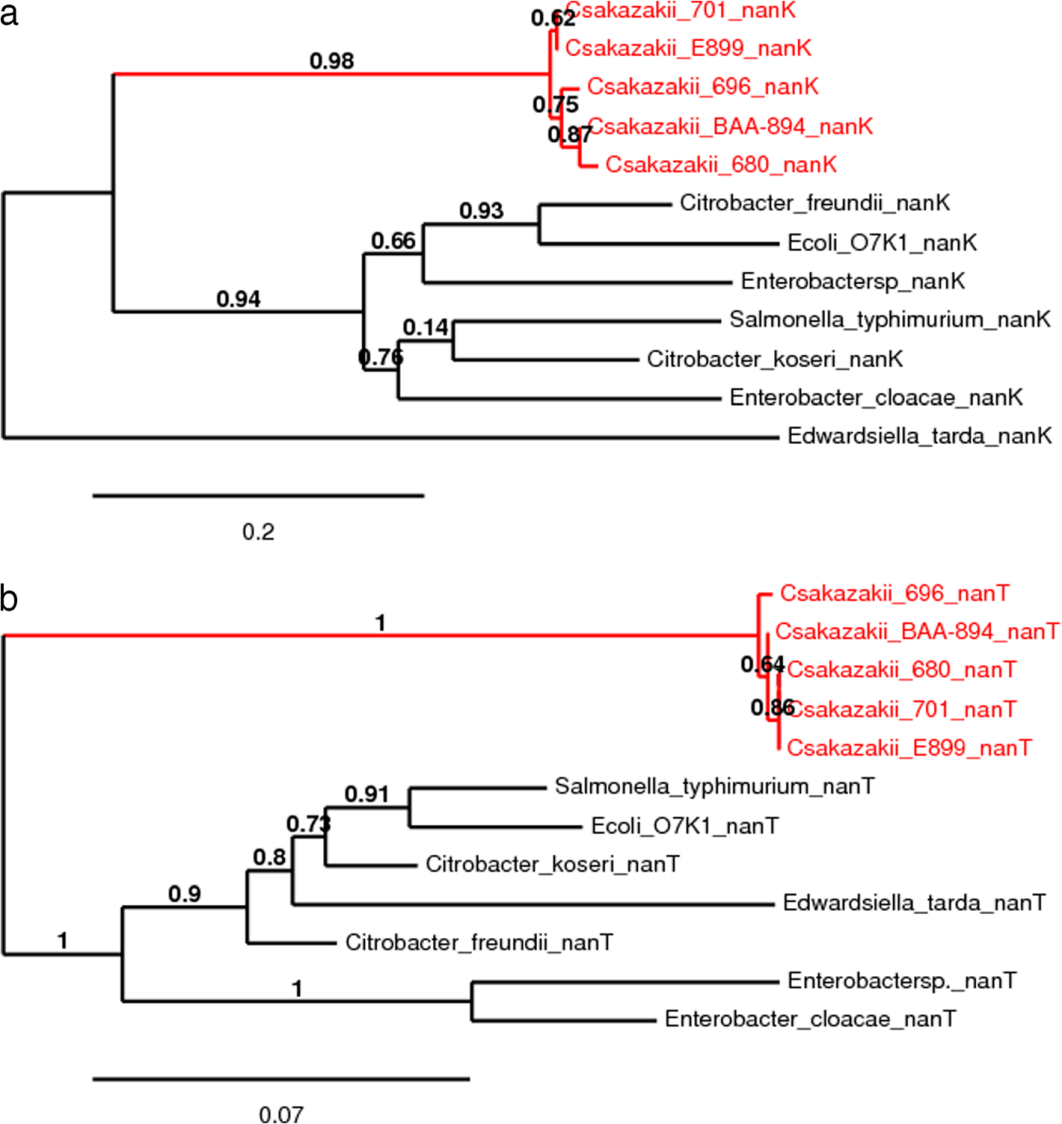
**Maximum-likelihood tree of (a) the NanA protein sequences (292 aa) and (b) NanR protein sequences (260 aa) of *C. sakazakii *and related *Enterobacteriaceae *members, constructed using PhyML, with 1000 bootstrap replicates.**

**Figure 2:**
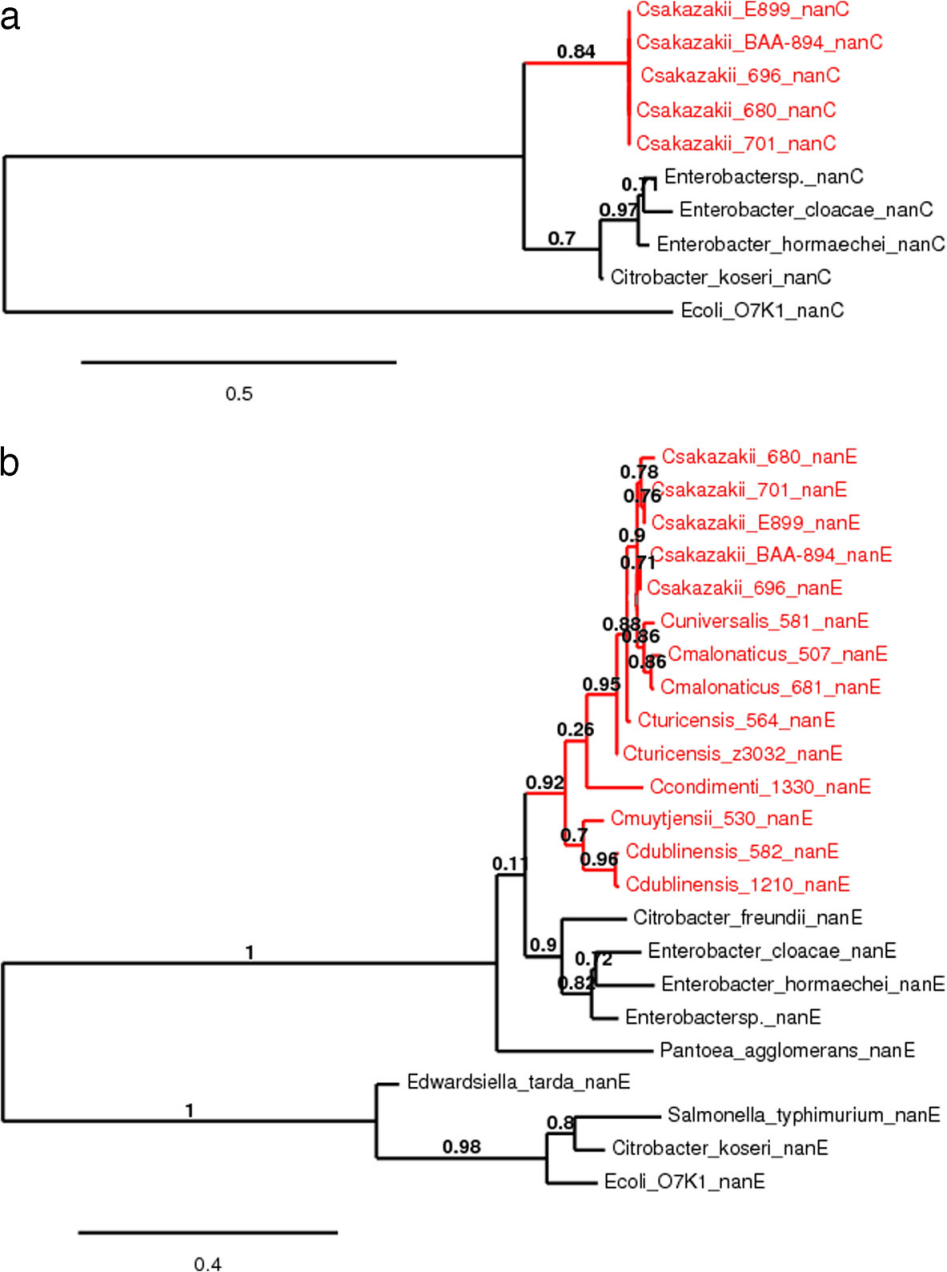
**Maximum-likelihood tree of (a) the NanK protein sequences (291 aa) and (b) NanT protein sequences (496 aa) of *C. sakazakii *and related *Enterobacteriaceae *members, constructed using PhyML, with 1000 bootstrap replicates.**

**Figure 3:**
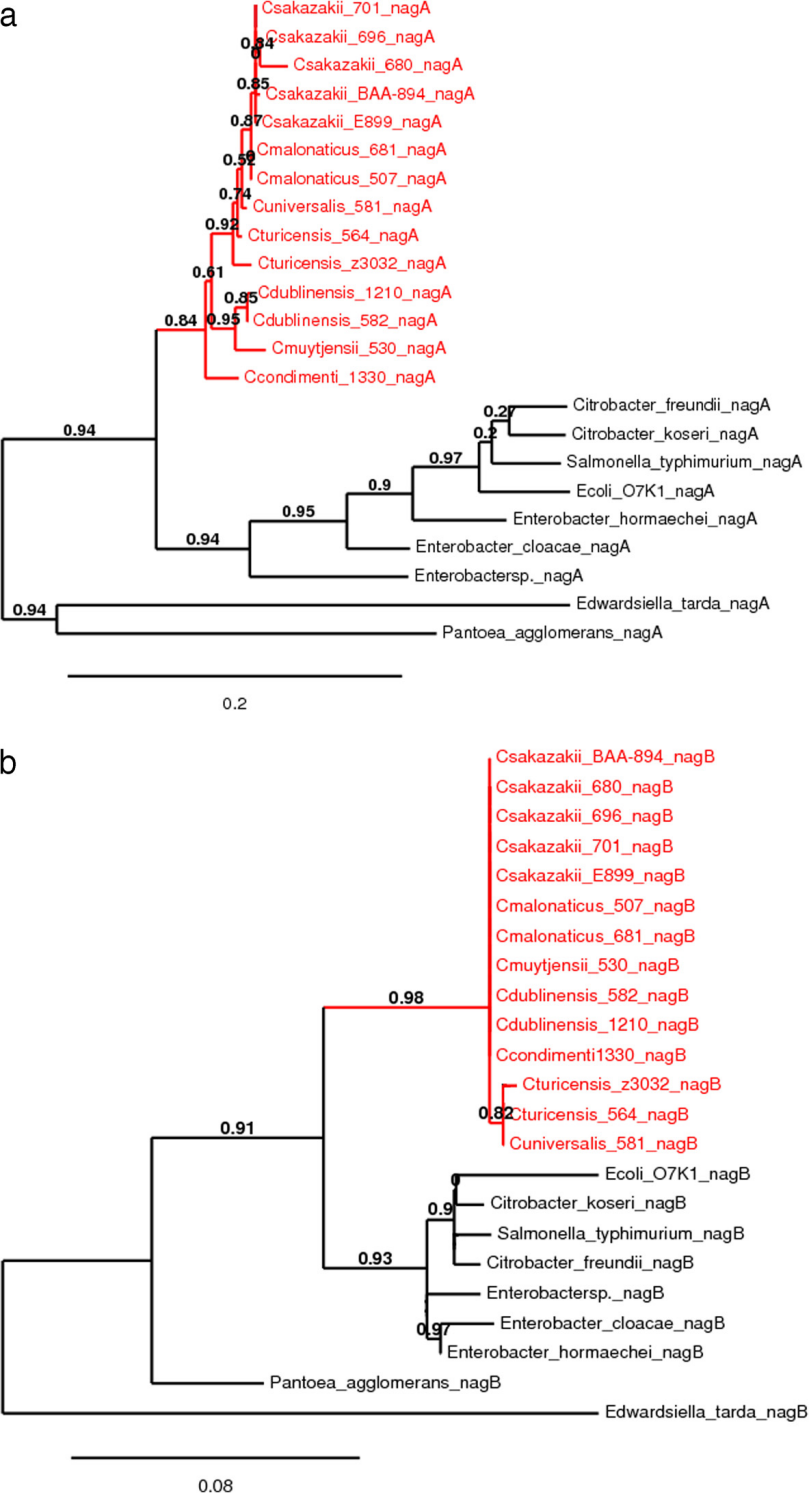
**Maximum-likelihood tree of (a) the NanE protein sequences (229 aa) and (b) NanC protein sequences (202 aa) of *Cronobacter *spp. and related *Enterobacteriaceae *members, constructed using PhyML, with 1000 bootstrap replicates.**

**Figure 4:**
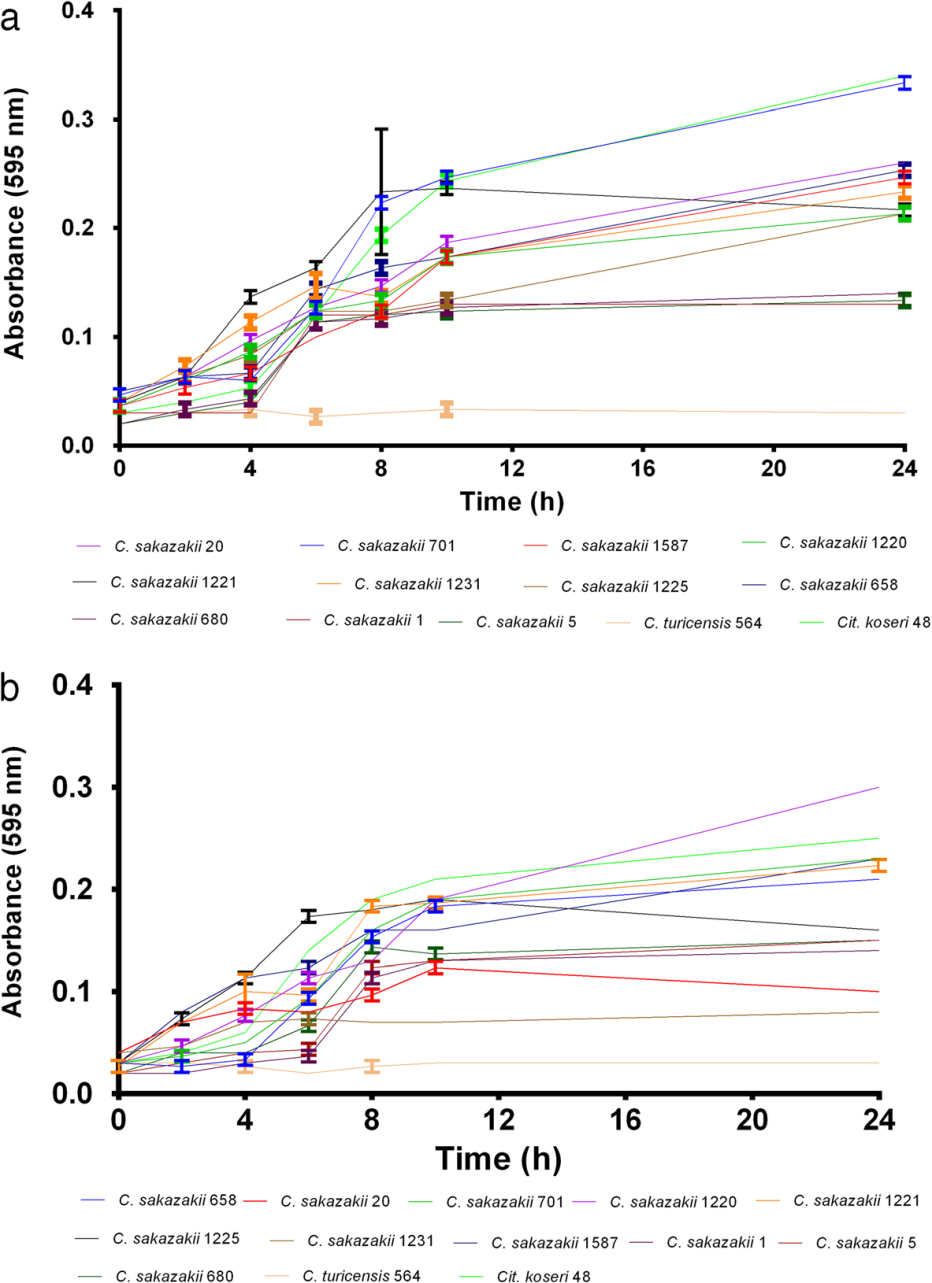
**Maximum-likelihood tree of (a) the NagA protein sequences (382 aa) and (b) NagB protein sequences (266 aa) of *Cronobacter *spp. and related *Enterobacteriaceae *members, constructed using PhyML, with 1000 bootstrap replicates.**

The predicted amino acid sequences of the proteins encoded by these *nan* cluster genes were analysed and their phylogenetic relationships with closely related Gram-negative bacteria is shown in Figures 1, 2, 3 and 4. Each predicted protein sequence from *C. sakazakii* formed an independent cluster, with the other *Enterobacteriaceae* members clustering on the neighbouring branches. In the NanA and NanR (Figure 1) phylogenetic trees, the *C. sakazakii* cluster appeared to evolve on the same branch as *E. cloacae* and *Enterobacter* spp., with the others forming a separate clade. In comparison, the NanK and NanT (Figure 2) *C. sakazakii* clusters appear to have greater phylogenetic distance from the other enteric members, with a clear split of the population into two clades, one of them being that of the *C. sakazakii* cluster. The *nanE* gene is found across the *Cronobacter* genus, and the phylogenetic analysis of the NanE protein sequences (Figure 3) revealed the *Cronobacter* cluster to have a common and closely related evolutionary clade with *E. cloacae, E. hormaechei, Enterobacter* spp., *Cit. freundii* and *Pantoea agglomerans.* The NanC protein in the *C. sakazakii* genomes could be located with >50% homology only in the genomes of *E. cloacae, E. hormaechei, Cit. koseri* and *E. coli* K1. Of these, the *E. coli* K1 NanC appeared to be very distantly related to the rest of the population studied (Figure 3). Both the *nagA* and *nagB* genes were found in the genomes of all the *Cronobacter* species (Figure 4). Phylogenetic analysis of these proteins showed the *Cronobacter* spp. sequences formed a distinct clade, with other *Enterobacteriaceae* members forming a neighbouring clade, both with a common evolutionary lineage*.* The newly identified species *C. condimenti* always branched within the *Cronobacter* genus cluster (Figures 3 and 4). Phylogenetic analysis of *Cit. koseri* and *Cit. freundii nan* gene sequences revealed different patterns in their branching, which indicates possible independent evolutionary paths for the *nan* genes within the *Citrobacter* genus.

### Growth of *C. sakazakii* on sialic acid and GM1

**Figure 5:**
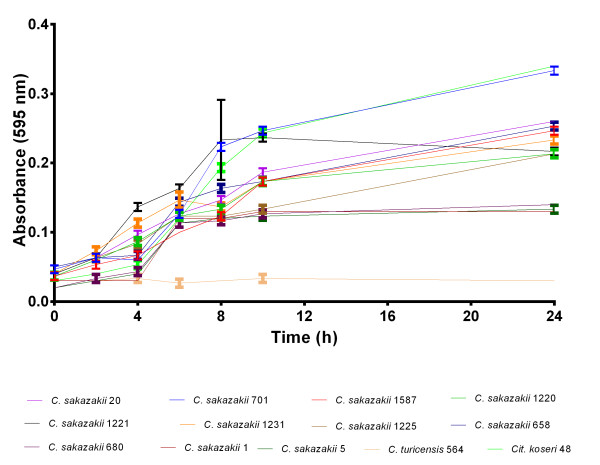
**Growth of *Cronobacter sakazakii *in M9 minimal medium supplemented with (a) sialic acid, and (b) GM1 ganglioside as sole carbon source.**

The sialic acid utilization pathway was confirmed in 6 strains of *C. sakazakii* by their growth in minimal medium (M9) with sialic acid as the sole carbon source; Figure 5. There was no growth by any strains (n=8) of the other six *Cronobacter* species. *Cit. koseri, Cit. freundii* and *Ed. tarda* which also have predicted *nan* genes grew in M9 supplemented with sialic acid. Growth was also observed for *C. sakazakii* strains, and *Cit. koseri* in minimal medium supplemented with the monosialoganglioside GM1 indicating possible sialidase activity; Figure 5 See Additional file 1: Table S1. No growth was observed for any organisms in M9 in the absence of a carbon source. Since the sialidase gene *nanH* had not previously been described for either of *C. sakazakii* or *Cit. koseri*, a bioinformatics re-analysis of their genomes was undertaken.

### Candidate sialidase genes in *C. sakazakii*

As in previous studies, BLAST searches of the *Cronobacter* genomes for homology to the well characterised sialidase gene (*nanH*) from *Haemophilus influenzae* and *Vibrio cholerae* did not result in any matches. However given the low homology (<30%) between sialidases the *Cronobacter* genomes were further investigated for the characteristic sialidase RIP and Asp box motifs [13]. The Asp box motif is composed of repeated Ser/Thr-×-Asp-×-Gly-×-Thr-Trp/Phe regions, where × represents any amino acid. No candidate genes were found encoding the RIF region and Asp box motifs in the *C. sakazakii* genome.

## Discussion

*C. sakazakii* is estimated to have branched from the rest of the *Cronobacter* members approximately 15–23 MYA [6], and is the *Cronobacter* species most frequently isolated from clinical infections of neonates. The absence of the core sialic acid utilization genes in the *Cronobacter* genus except for *C. sakazakii,* suggests their acquisition was by horizontal gene transfer instead of gene loss by the rest of the genus. The metabolism of sialic acid could have a role in the organisms’ virulence. The evolution of *C. sakazakii* post-dates key eras in the evolution of sialic acid biosynthetic pathway which occurred near the divergence of Coelomata (protostomes and deuterostomes *ca.*500 MYA), and milk producing mammals (Jurassic Era *ca*.200-145 MYA). Therefore the presence of sialic acid is a relatively recent evolution, and high levels are accessible to bacteria in milk, the intestines, and the brain.

The *C. sakazakii* genomes possess the genes required for the uptake and catabolism of sialic acid; Table 1. Although the *nanAKTER* genes are usually clustered together, in *C. sakazakii nanE* is located at a different region (ESA_00529) from the others (ESA_03610-12). This split has also been reported for *Ed. tarda* and *Cit. freundii*[14]. This possibly reflects a separate evolutionary lineage for the gene*.* All the *C. sakazakii* genomes encode for the NanC outer membrane porin and NanT inner membrane transporter protein. Hence, all *C. sakazakii* strains can potentially transport the exogenous sialic acid into the cytoplasm of their cells. Interestingly, all the *Cronobacter* species genomes also exhibited the presence of the genes (*siaPQM*) encoding for the TRAP transporter; Table 1. However laboratory growth studies confirmed that *C. sakazakii* was the only *Cronobacter* species which could grow on sialic acid as a carbon source and indicates that NanT is the active transporter in the process; Figure 5a*.* The acquisition of genes encoding for the utilization of exogenous sialic acid may have a major role in *C. sakazakii* colonisation of the human intestinal tract (via mucins) and the use of sialic acid in breast milk or infant formula as a nutrient source [9,18].

The demonstration of *C. sakazakii* growth on the ganglioside GM1 (Figure 5b) indicated the possible possession of a sialidase enzyme, which despite previous sequence searches for *nanH* (the gene encoding for sialidase) had not been reported. Since the homology between sialidases is very low (<30%) [13], a more detailed search for the sialidase RIP and Asp-box motifs was undertaken. However no candidate genes were found with both RIP and Asp-box motifs. Nevertheless the growth on GM1 indicated a ganglioside degradative ability by *C. sakazakii*. GM1 is composed of galactose, N-acetyl-galactose, glucose as well as sialic acid units linked by β 1–3 and β 1–4 linkages, and these are joined to a steroid. Therefore it is proposed that GM1 is degraded to metabolisable sugar units by the activity of various β-galactosidases (ESA_01827, ESA_02977 & ESA_03417), β-acetyl-hexosaminidases (ESA_02237 and ESA_02655), esterases (ESA_00377 & ESA_00776) and lipases (ESA_02127 & ESA_02202), and therefore able to grow on this substrate.

The %GC content of the *nanC* gene was found to be 47%, considerably less than the 56% GC content of the overall *Cronobacter* genome. Slight aberrations were also seen in the %GC content values of the *nanK* and *nanE* genes, an observation previously noted in an evolutionary study of the *nan* clusters of other *Enterobacteriaceae* members such as *Salmonella enterica, E. coli* and *Yersinia* spp. [11]. These observations suggest a strong possibility of horizontal transfer events having influenced separate acquisitions for the *nanAKT* cluster in *C. sakazakii,* as well as the *nanC* and *nanE* clusters in the whole genus. Hence, it is possible that the *nan* clusters could have evolved in a mosaic pattern in this bacterial genus. A phylogenetic analysis was also conducted using the protein sequences encoded by the sialic acid utilization genes in *C. sakazakii* (Figures 1, 2, 3 and 4). This revealed that the *nanATKR* genes have evolved as a lineage in *C. sakazakii*, and independent of other closely related pathogens of the *Enterobacteriaceae* family. No close homology (50% cut-off) was found between the NanC protein sequences and those from most other members of the *Enterobacteriaceae*. This indicates the acquisition of the associated gene in *C. sakazakii* was from a more distantly related organism, currently unidentified.

It is also notable that the *nanATK* cluster in *C. sakazakii* is located adjacent to a stringent starvation gene homologue (*sspA*, ESA_03615) and therefore expression of this gene cluster could be responsive to environmental nutrient levels; Table 1. The presence of other related genes such as *nagA* and *nagB,* leading to the synthesis of fructose-6-phosphate, support the role of sialic acid as a carbon source in *C. sakazakii.* Whereas the absence of genes such as *neuS, neuO* and *neuD* in the genomes of the *Cronobacter* genus indicates that the organism does not decorate its cell surface with sialic acid, unlike *E. coli* K1 and *N. meningitidis*[10,14]*.*

## Conclusions

Three rich mammalian sources of sialic acid for pathogenic or commensal bacteria are in the gastrointestinal tract, the brain and in human milk. These three locations of sialic acid correlate with the clinical presentations of *C. sakazakii* infections with respect to necrotizing enterocolitis and the intensive brain damage during neonatal meningitis. This is especially pertinent since most of the neonatal infections in *C. sakazakii* have been reported at a very early stage of growth with half in the first week and three quarters during the first month of age [1,2,4]. Apart from these mammalian sources of sialic acid, another feature with the organism is that sialic acid is often added to infant formula, a recognised source of neonatal *Cronobacter* infections [4,6]. The unique utilization of sialic acid by *C. sakazakii* indicates a plausible adaptation of the organism to host. The source of the *nan* genes is not fully understood, but appears to be by multiple horizontal gene transfers, the sources of which are uncertain to date.

## Methods

### Strains and culture conditions

Nineteen *Cronobacter* strains were selected which represented the seven recognized species, and included those from reported clinical cases and species type strains (Table 2). All *Cronobacter* strains were stored at −80°C in Nutrient Broth (Oxoid, UK) with 10% glycerol, subcultured on Trypticase Soy Agar (Oxoid ThermoFisher, UK) and checked for purity. *Cit. koseri* strain 48, *Cit. freundii* 1927, *Ed. tarda* 1926 were included for comparative purposes.

**Table 2:** Bacterial strains used for laboratory studies of growth on sialic acid and GM1 as sole carbon source

**Organism**	**Strain**	**Sequence type**^**a**^	**Source**
*C. sakazakii*	1^b^	ST8	Throat
*C. sakazakii*	5	ST8	Blood
*C. sakazakii*	658	ST1	Non-infant formula
*C. sakazakii*	680	ST8	Cerebral spinal fluid
*C. sakazakii*	696	ST12	Faeces
*C. sakazakii*	701	ST4	Peritoneal fluid
*C. sakazakii*	1220	ST4	Cerebral spinal fluid
*C. sakazakii*	1221	ST4	Cerebral spinal fluid
*C. sakazakii*	1225	ST4	Blood
*C. sakazakii*	1231	ST4	Faeces
*C. sakazakii*	1587	ST4	Cerebral spinal fluid
*C. malonaticus*	507	ST11	Faeces
*C. malonaticus*	681^c^	ST7	Breast abscess
*C. universalis*	581	ST54	Water
*C. turicensis*	564	ST5	Blood
*C. turicensis*	1211^d^	ST19	Blood
*C. muytjensii*	530	ST49	Powdered infant formula
*C. dublinensis*	582	ST36	Unknown
*C. condimenti*	1330^e^	ST40	Spiced sausages
*Ed. tarda*	1926	-	Unknown
*Cit. freundii*	1927	-	Unknown
*Cit. koseri*	48	-	Clinical

^a.^ Sequence type as according to the *Cronobacter* genus multilocus sequence typing scheme database; http://www.pubMLST.org/cronobacter.

^b.^*C. sakazakii* species type strain (ATCC 29544^T^).

^c.^*C. malonaticus* species type strain (LMG 23826^T^).

^d.^*C. turicensis* species type strain (LMG 28327^T^).

^e.^*C. condimenti* species type strain.

### Sialic acid utilization and sialidase degradation determination

The method of Almagro-Moreno and Boyd [11] was followed with two modifications. The bacterial cultures were grown overnight in Brain Heart Infusion broth (Oxoid ThermoFisher, UK), and the cell suspension was washed three times before the assay to remove the carryover of nutrients. Growth in M9 minimal media with sialic acid (1 mg/ml, Sigma Aldrich), or GM1 monosialoganglioside (1 mg/ml, Sigma Aldrich) as the carbon source was measured by absorbance increase at 595 nm. Inoculation of M9 without a carbon source was used as a negative control, and glucose was added (1 mg/ml) to the minimal medium as a positive control.

### Phylogenetic analysis of sialic acid utilization genes

The amino acid sequences of the genes *nanK, nanT, nanE, nanA, nanC, nagA* and *nagB* from the fourteen *Cronobacter* spp. genomes (CALG01000001-C201, CALF01000001-569, CALE01000001-768, AFMO00000000, NC_009778-80, CALD01000001-249, CALC01000001-171, CALB01000001-114, NC_013282-85, CALA01000001-427, CAKZ01000001-221, CAKY01000001-365, CAKX01000001-231, CAKW01000001-155) were obtained from RAST prokaryotic genome annotation server. The gene sequences from the genome of *C. sakazakii* BAA-894 was used to perform tblastx searches to obtain the corresponding sequences of closely related organisms showing >50% amino acid identity. The corresponding protein sequences were downloaded from the genomes of *E. coli* O7:K1 strain CE10 (Accession No. NC_017646); *E. cloacae* subsp. *cloacae* ATCC 13047 (Accession No. NC_014121); *E. hormaechei* ATCC 49162 (Accession No. AFHR00000000); *Enterobacter* spp. strain 683 (Accession No. CP000653); *Cit. freundii* strain 4_7_47CFAA (Accession No. ADLG00000000); *Cit. koseri* ATCC BAA-895 (Accession No. NC_009792); *Ed. tarda* strain EIB202 (Accession No. NC_013508); *Salmonella enterica* subsp. *enterica* serovar Typhimurium strain LT2 (Accession No. NC_003197); *Pantoea agglomerans* strain IG1 (Accession No. BAEF00000000). The sequences of each gene were aligned using ClustalW [19] and phylogenetic analysis was performed using the Maximum-Likelihood algorithm in PhyML [20,21]. Stability of the relationships was assessed by the bootstrap method (1000 replicates) and the trees were viewed and annotated using TreeDyn at http://www.phylogeny.fr[22,23].

## Competing interests

The authors declare that they have no competing interests.

## Authors’ contributions

SJ and NM carried out the genome sequence analysis. SH undertook the laboratory studies. SH and SJF designed the laboratory studies. SJF conceived the study. SJ and SJF drafted the manuscript. All authors read and approved the final manuscript.

## Supplementary Material

Additional file 1: Table S1 Growth of *Cronobacter sakazakii* in M9 minimal medium supplemented with (a) sialic acid, and (b) GM1 ganglioside as sole carbon source. Click here for file
