# Supplementary material for: Sialic acid utilization by Cronobacter sakazakii
Source: Microb Inform Exp. 2013 May 24;3:3. doi: 10.1186/2042-5783-3-3 (PMC3716653; doi:10.1186/2042-5783-3-3)
Supplement: Additional file 1: Table S1 — Growth of Cronobacter sakazakii in M9 minimal medium supplemented with (a) sialic acid, and (b) GM1 ganglioside as sole carbon source. [file 2042-5783-3-3-S1.docx]

Supplementary Table 2a. Growth of *Cronobacter sakazakii* in M9 minimal medium supplemented with sialic acid as sole carbon source.

| Species | | *C. sakazakii* | | | | | | | *C. turicensis* | | *C. malonaticus* | | *C. condimenti* | *C. universalis* | *C. muytjensii* | *C. dublinensis* | *Cit. koseri* | *Cit. freundii* | *Ed. tarda* |
| --- | --- | --- | --- | --- | --- | --- | --- | --- | --- | --- | --- | --- | --- | --- | --- | --- | --- | --- | --- |
| Strain | | 658 | 701 | 1220 | 1221 | 1225 | 1587 | 1 | 564 | 1211 | 507 | 681 | 1330 | 581 | 530 | 582 | 48 | 1927 | 1926 |
| Sequence type^a^ | | ST1 | ST4 | ST4 | ST4 | ST4 | ST4 | ST8 | ST5 | ST19 | ST11 | ST7 | ST40 | ST54 | ST49 | ST36 |  |  |  |
|  | 0 | 0.05^b^ | 0.05 | 0.04 | 0.04 | 0.04 | 0.04 | 0.03 | 0.02 | 0.02 | 0.02 | 0.02 | 0.02 | 0.02 | 0.02 | 0.02 | 0.03 | 0.03 | 0.02 |
|  | 2 | 0.06 | 0.06 | 0.06 | 0.06 | 0.06 | 0.05 | 0.03 | 0.03 | 0.03 | 0.03 | 0.04 | 0.04 | 0.04 | 0.03 | 0.03 | 0.04 | 0.05 | 0.04 |
|  | 4 | 0.07 | 0.06 | 0.09 | 0.14 | 0.08 | 0.07 | 0.03 | 0.03 | 0.03 | 0.03 | 0.04 | 0.04 | 0.04 | 0.03 | 0.03 | 0.05 | 0.07 | 0.06 |
| Time (h) | 6 | 0.14 | 0.13 | 0.12 | 0.16 | 0.12 | 0.10 | 0.12 | 0.03 | 0.03 | 0.03 | 0.04 | 0.04 | 0.04 | 0.03 | 0.03 | 0.12 | 0.12 | 0.12 |
|  | 8 | 0.16 | 0.22 | 0.13 | 0.20 | 0.12 | 0.12 | 0.12 | 0.03 | 0.03 | 0.03 | 0.04 | 0.04 | 0.04 | 0.03 | 0.03 | 0.19 | 0.23 | 0.22 |
|  | 10 | 0.17 | 0.25 | 0.17 | 0.24 | 0.13 | 0.17 | 0.13 | 0.03 | 0.03 | 0.02 | 0.03 | 0.04 | 0.04 | 0.03 | 0.03 | 0.24 | 0.26 | 0.24 |
|  | 24 | 0.25 | 0.33 | 0.21 | 0.22 | 0.21 | 0.25 | 0.13 | 0.02 | 0.03 | 0.04 | 0.04 | 0.05 | 0.04 | 0.04 | 0.04 | 0.34 | 0.34 | 0.32 |

a Sequence type as according to the *Cronobacter* genus multilocus sequence typing scheme database; <http://www.pubMLST.org/cronobacter>.

b Absorbance at 595nm. Negative control was inoculated M9 medium without additional carbon source, absorbance values were 0.02-0.03U over the period of the experiment.

Supplementary Table 2b. Growth of *Cronobacter sakazakii* in M9 minimal medium supplemented with GM1 ganglioside as sole carbon source.

|  | Species | *C. sakazakii* | | | | | | | | | *C. turicensis* | *Cit. koseri* |
| --- | --- | --- | --- | --- | --- | --- | --- | --- | --- | --- | --- | --- |
|  | Strain | 658 | 701 | 1220 | 1221 | 1225 | 1587 | 1 | 5 | 680 | 564 | 48 |
|  | Sequence type^a^ | ST1 | ST4 | ST4 | ST4 | ST4 | ST4 | ST8 | ST8 | ST8 | ST5 |  |
|  | 0 | 0.03^b^ | 0.03 | 0.03 | 0.03 | 0.03 | 0.03 | 0.02 | 0.02 | 0.02 | 0.03 | 0.03 |
|  | 2 | 0.03 | 0.04 | 0.05 | 0.07 | 0.07 | 0.08 | 0.02 | 0.03 | 0.04 | 0.03 | 0.04 |
|  | 4 | 0.03 | 0.05 | 0.07 | 0.08 | 0.11 | 0.11 | 0.03 | 0.04 | 0.04 | 0.03 | 0.06 |
| Time (h) | 6 | 0.09 | 0.10 | 0.11 | 0.10 | 0.17 | 0.12 | 0.04 | 0.04 | 0.07 | 0.02 | 0.14 |
|  | 8 | 0.15 | 0.16 | 0.13 | 0.18 | 0.18 | 0.16 | 0.11 | 0.12 | 0.14 | 0.03 | 0.19 |
|  | 10 | 0.18 | 0.19 | 0.19 | 0.19 | 0.19 | 0.16 | 0.13 | 0.13 | 0.14 | 0.03 | 0.21 |
|  | 24 | 0.21 | 0.23 | 0.30 | 0.22 | 0.16 | 0.23 | 0.14 | 0.15 | 0.15 | 0.03 | 0.25 |

a Sequence type as according to the *Cronobacter* genus multilocus sequence typing scheme database; <http://www.pubMLST.org/cronobacter>.

b Absorbance at 595nm. Negative control was inoculated M9 medium without additional carbon source, absorbance values were 0.02-0.03U over the period of the experiment.
